# A Narrative Review of AI Frameworks for Chronic Stress Detection Using Physiological Sensing: Resting, Longitudinal, and Reactivity Perspectives

**DOI:** 10.3390/s26082345

**Published:** 2026-04-10

**Authors:** Totok Nugroho, Wahyu Rahmaniar, Alfian Ma’arif

**Affiliations:** 1 Department of Information and Communications Engineering, Institute of Science Tokyo, Yokohama 226-8501, Japan; totok.nugroho@mail.ugm.ac.id; 2 Institute of Integrated Research, Institute of Science Tokyo, Yokohama 226-5801, Japan; 3 Department of Electrical Engineering, Universitas Ahmad Dahlan, Yogyakarta 55191, Indonesia; alfianmaarif@ee.uad.ac.id

**Keywords:** chronic stress, physiological sensing, electroencephalography (EEG), heart rate variability (HRV), photoplethysmography (PPG), electrodermal activity (EDA), wearable sensors, machine learning (ML), deep learning (DL), multimodal fusion

## Abstract

Chronic stress is a time-dependent condition characterized by sustained dysregulation across neural, autonomic, and endocrine systems, with important consequences for both health and socioeconomic outcomes. Unlike acute stress, which is typically characterized by short-lived physiological activation, chronic stress reflects an accumulated allostatic load and a longer-term recalibration of stress response systems. Recent advances in physiological sensing and artificial intelligence (AI) have supported the development of computational approaches for chronic stress detection using electroencephalography (EEG), heart rate variability (HRV), photoplethysmography (PPG), electrodermal activity (EDA), and wearable multimodal platforms. This narrative review examines current AI-based studies through three main inferential paradigms: resting baseline dysregulation, longitudinal physiological monitoring, and reactivity-based inference. Across modalities, classical machine learning (ML) methods, particularly support vector machines (SVMs) and tree-based ensembles, remain the most commonly used approaches, largely because available datasets are small and most pipelines still depend on engineered features. Deep learning (DL) methods are beginning to emerge, but their use remains constrained by the lack of large, standardized, longitudinal datasets specifically designed for chronic stress research. Major challenges include ambiguity in stress labeling, limited longitudinal validation, circadian confounding, inter-individual variability, and small cohort sizes. Future progress will depend on standardized datasets, biologically grounded multimodal integration, hybrid baseline-reactivity modeling, adaptive personalization, and more interpretable AI systems. Greater emphasis is also needed on clinical relevance and generalizability if AI-based chronic stress monitoring is to move beyond experimental settings.

## 1. Introduction

Chronic stress and related mental health problems affect more than one billion people worldwide and continue to impose major health, social, and economic burdens. Anxiety and depression are recognized by the World Health Organization as leading contributors to long-term disability and are associated with substantial health-care costs and productivity loss [1]. Mental ill-health linked to prolonged stress exposure can also reduce workforce participation and has been estimated to account for up to 4% of gross domestic product in many countries [2]. Chronic stress should therefore be regarded not only as an individual clinical concern, but also as a broader public health and societal challenge.

From a biological perspective, chronic stress develops when environmental demands persist over time and exceed adaptive capacity, resulting in dysregulation across neuroendocrine, autonomic, immune, and neural systems [3]. Acute stress typically involves transient activation of the autonomic nervous system and the hypothalamic–pituitary–adrenal (HPA) axis, followed by recovery. Chronic stress, in contrast, is associated with longer-term maladaptive changes in regulatory function that accumulate as allostatic load, often described as the biological wear and tear produced by repeated stress exposure [4,5]. Increased allostatic load has been linked to cardiovascular disease, metabolic dysfunction, depression, cognitive decline, and premature mortality [6]. Chronic stress should therefore not be understood simply as prolonged acute activation but as a condition marked by persistent baseline shifts, reduced physiological flexibility, and altered responses to subsequent challenges.

Despite its importance, chronic stress remains difficult to assess accurately. Self-report instruments such as the Perceived Stress Scale (PSS) and the Trier Inventory for Chronic Stress (TICS) are widely used to estimate stress over extended periods. While they serve as valuable proxies for accumulated allostatic load, they are not equivalent to clinical diagnoses and remain subjective and vulnerable to recall bias [7,8]. Biochemical indicators, such as salivary cortisol, are more informative for short-term HPA-axis activity and are strongly influenced by circadian timing and situational context [9]. For this reason, increasing attention has been directed toward physiological markers that may better reflect cumulative dysregulation, including hair cortisol concentration (HCC) as a retrospective index of long-term cortisol secretion [10,11], reduced HRV as a marker of impaired autonomic flexibility [12,13], and altered cortical dynamics measurable with EEG [14,15].

Because chronic stress develops over time, its detection must capture sustained dysregulation, longitudinal trends, and altered reactivity rather than brief stimulus-driven responses. Conventional statistical approaches are often limited in their ability to model such complex, nonlinear, and multimodal relationships. AI, including ML and DL, offers a promising framework for integrating physiological data, such as EEG, HRV, electrodermal activity, and signals from wearable sensors, to detect latent patterns associated with prolonged dysregulation [16,17,18,19]. However, current studies remain highly heterogeneous in their designs, labeling strategies, validation protocols, and model interpretability. Previous reviews have largely focused on acute stress or general psychological stress, often blurring the distinction between transient stress responses and the cumulative physiological burden of chronic stress. As a result, methods for modeling long-term dysregulation are rarely discussed separately from methods for detecting momentary stress spikes.

This narrative review addresses this overlap by explicitly isolating chronic stress as a distinct physiological and computational challenge. To distinguish the present work from previous literature, the main contributions are outlined as follows:

- Shifting the analytical focus: This review shifts the focus away from brief, stimulus-induced responses (i.e., acute stress) to emphasize the necessity of modeling sustained baseline abnormalities, longitudinal physiological changes, and altered stress reactivity (i.e., chronic stress).
- A novel paradigm-based synthesis: Current AI-based approaches are categorized into three distinct, time-dependent inferential paradigms that specifically reflect chronic dysregulation—resting baseline analysis, longitudinal monitoring, and reactivity-based inference.
- Biological and clinical evaluation: Existing ML and DL pipelines are critically evaluated against the biological realities of allostatic load, identifying the specific steps required to transition these systems from controlled experiments to real-world applications.

The remainder of this article is structured as follows. The methodology used to synthesize the literature is outlined in Section 2. The conceptual and biological foundations of chronic stress, along with current labeling frameworks and the three major inferential paradigms, are detailed in Section 3. The primary physiological sensing modalities and AI methods employed in the field are examined in Section 4 and Section 5, respectively. Persistent methodological challenges are addressed in Section 6, followed by an analysis of emerging research gaps and future directions in Section 7. Finally, the concluding remarks are provided in Section 8.

## 2. Methodology

This study was conducted as a narrative review. The objective was to identify and synthesize existing research on AI-based chronic stress detection using physiological sensing, with a specific focus on sensing modalities, inferential tasks, and analytical approaches. While this is a narrative review rather than a formal systematic review, a structured search strategy was applied to ensure that the literature selection process was transparent and unbiased.

### 2.1. Literature Search and Selection Strategy

This narrative review employed a targeted literature search focusing on the intersection of AI, physiological sensing, and chronic stress. The search strategy used specific keyword combinations across three conceptual groups: stress type, inferential task, and physiological modality, as detailed in Table 1. To broaden coverage across engineering, biomedical, and interdisciplinary literature, the search was conducted in Scopus, PubMed, and Web of Science. Because the review specifically emphasizes AI-based chronic stress inference, study selection was purposively restricted to papers that explicitly described an automated computational framework, such as ML classification, regression, clustering, or DL pipelines applied to physiological data. This ensured that the review remained focused on algorithmic stress detection and monitoring, rather than on purely clinical, psychological, or endocrinological discussions without an AI component.

### 2.2. Summary of Included Studies

The final synthesis comprised 23 studies. Consistent with the technical scope of this review, most included studies focused on computational stress inference from physiological signals using ML or related AI approaches. Among the 23 included studies, EEG was the most frequently represented modality, appearing in 12 studies (52.2%). Multimodal approaches accounted for 7 studies (30.4%), while wearable or ambulatory monitoring designs were used in 8 studies (34.8%). Methodologically, the literature was dominated by classical ML approaches, which appeared in 18 studies (78.3%), whereas neural-network-based methods were used in 5 studies (21.7%).

### 2.3. Scope and Limitations of the Search Strategy

It is important to acknowledge the boundaries of this targeted narrative search. Because this study is structured as a narrative review rather than a formal systematic review, we acknowledge that the evidential rigor is naturally more limited. Although the use of Scopus, PubMed, and Web of Science broadens disciplinary coverage, the review intentionally prioritizes studies that explicitly implement AI-based or automated inferential pipelines for chronic stress detection. As a result, the synthesis is naturally weighted toward engineering, biomedical signal processing, wearable sensing, and computational modeling. Consequently, while the biological and clinical context of chronic stress and allostatic load is considered where relevant, this review does not aim to exhaustively cover purely clinical, psychiatric, endocrinological, or behavioral studies that do not include a computational inference component. This focused search boundary was adopted to maintain a clear emphasis on the models, physiological features, sensing modalities, and validation strategies that underpin AI-driven chronic stress detection.

## 3. Conceptual Foundations of Chronic Stress

### 3.1. Stress Across Timescales: Acute Response, Chronic Dysregulation, and Allostatic Load

**Figure 1:**
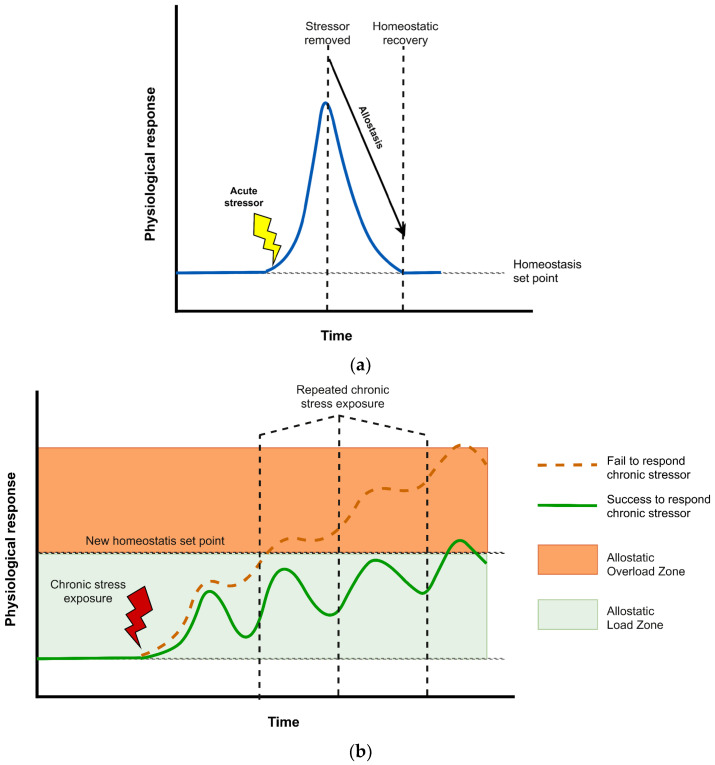
Acute vs. chronic stress responses: (**a**) Temporary acute stress response and recovery; (**b**) Under chronic stress, successful adaptation (solid green line) maintains dynamic responsiveness at a new set point, while failed adaptation (dashed orange line) results in allostatic overload and a blunted response.

Stress is generally understood as an adaptive biological response that enables the organism to cope with environmental demands through coordinated neuroendocrine and autonomic activation. As shown in Figure 1a, exposure to an acute stressor activates the sympathetic–adrenomedullary (SAM) system and the HPA axis, leading to the release of catecholamines and glucocorticoids such as cortisol [3,20]. Through this process, energy is mobilized, vigilance is increased, and short-term physiological adjustments are produced to support survival. Under normal conditions, homeostasis is restored after the stressor has passed, via negative feedback mechanisms largely mediated by glucocorticoid receptor signaling [20,21]. This recovery phase reflects adaptive regulation and is consistent with the concept of allostasis, in which stability is maintained through physiological change.

A different pattern is observed under chronic stress. When stressors are repeated, prolonged, or experienced as uncontrollable, stress -response systems may remain activated without adequate recovery [3,4]. As illustrated in Figure 1b (adapted from [22]), repeated activation gradually shifts physiological regulation away from the original homeostatic set point, indicating the accumulation of allostatic load. Individuals who successfully respond to chronic stressors (indicated by the solid green line) demonstrate baseline adaptation, establishing a new homeostatic set point within the allostatic load zone without reaching overload. Notably, their physiological response maintains dynamic fluctuations over time rather than becoming blunted. In contrast, a failure to adequately respond to chronic stressors (indicated by the dashed orange line) drives the system into allostatic overload, characterized by a severely dysregulated and blunted physiological response. Chronic stress should therefore not be viewed simply as an extended version of acute stress. Rather, it involves a broader recalibration of regulatory mechanisms across interconnected neuroendocrine, cardiovascular, immune, and neural systems [5]. With prolonged glucocorticoid exposure, receptor sensitivity may be impaired, and HPA-axis feedback regulation may become less efficient, resulting in altered cortisol rhythms, reduced adaptability, and poorer baseline control [23,24]. Chronic stress is not always expressed as persistently elevated cortisol. In many cases, it appears instead as maladaptive secretion patterns or reduced physiological flexibility in response to later challenges [23].

The distinction between acute and chronic stress becomes clearer within the framework of allostasis, which describes how stability is achieved through dynamic physiological adjustment [4]. Although allostatic responses are beneficial in the short term, repeated or sustained activation produces a cumulative biological burden referred to as allostatic load [5]. This burden has been quantified using composite indices that include neuroendocrine markers, inflammatory mediators, metabolic indicators, and cardiovascular measures [6,25]. Elevated allostatic load has been associated with increased morbidity, functional decline, and mortality, highlighting the long-term consequences of sustained stress exposure [25].

From this perspective, chronic stress is a time-dependent condition marked by sustained multisystem dysregulation and accumulated allostatic load [22], rather than as a simple prolongation of acute stress. This dysregulation is evident not only in resting-state abnormalities but also in altered acute stress reactivity, in which physiological responses may become blunted, exaggerated, or poorly regulated. For physiological sensing research, this means focusing on persistent baseline abnormalities, longitudinal change, and maladaptive reactivity rather than brief stimulus-bound responses.

### 3.2. Biological Signatures of Sustained Dysregulation

**Figure 2:**
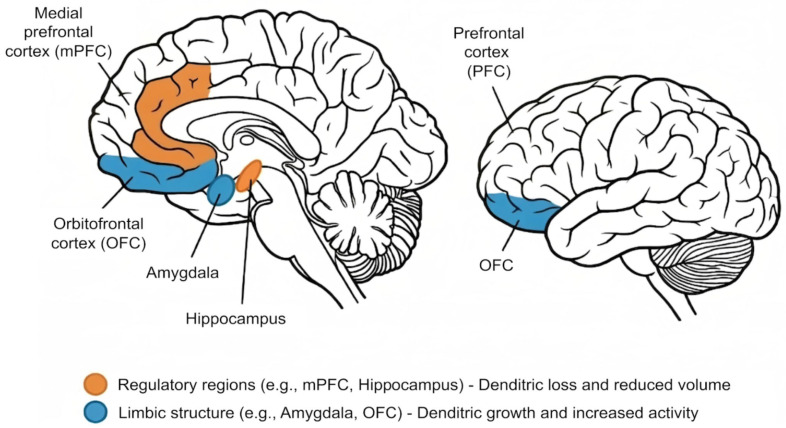
Neural structural changes associated with the transition from acute to chronic stress.

Chronic stress is expressed through measurable changes in neural, autonomic, and endocrine functions, collectively reflecting a sustained regulatory imbalance. At the neural level, prolonged exposure to stressors has been associated with structural remodeling in stress-sensitive brain regions. As shown in Figure 2, chronic stress is associated with dendritic loss and reduced volume in regulatory regions such as the prefrontal cortex and hippocampus, whereas dendritic growth and heightened activity have been reported in limbic regions, including the amygdala and orbitofrontal cortex [22]. These changes suggest a shift away from adaptive acute stress regulation toward a chronic state in which top-down executive control is weakened and emotional reactivity becomes more prominent. As a result, cognitive flexibility, emotional regulation, and coping capacity may be compromised, thereby increasing vulnerability to continued exposure to stress [24,26]. At the functional level, chronic stress has also been associated with altered cortical oscillations and disrupted network connectivity, providing a neurophysiological basis for EEG-based detection approaches.

Within the autonomic domain, chronic stress has consistently been associated with reduced HRV, which reflects diminished parasympathetic modulation and impaired neurovisceral integration [27]. Lower HRV indicates reduced physiological flexibility and has been linked to increased cardiovascular risk and broader stress-related morbidity [28]. Thus, chronic stress is characterized not only by transient sympathetic activation but also by altered baseline autonomic balance, suppressed variability, and impaired recovery after stress exposure.

**Figure 3:**
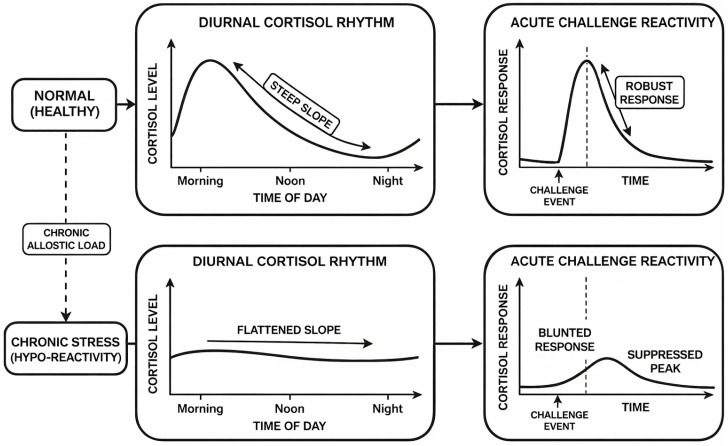
Endocrine adaptation to chronic stress is characterized by a flattened diurnal cortisol rhythm and a hypo-reactive stress response.

Endocrine changes are primarily reflected in dysregulation of the HPA axis. Prolonged stress exposure may alter circadian cortisol rhythms, producing flatter diurnal slopes and blunted responses to acute challenges [23,24], as illustrated in Figure 3. Such patterns suggest recalibrated feedback sensitivity and longer-term adaptation of stress response systems. Notably, chronic stress is not always accompanied by exaggerated physiological responses. Under sustained load, attenuated or hypo-reactive responses may also emerge, which is consistent with models of stress-related physiological downregulation [23].

Taken together, these biological signatures, including altered cortical dynamics, reduced autonomic flexibility, and dysregulated endocrine rhythms, indicate that chronic stress is a systemic condition associated with accumulated allostatic load rather than merely a prolonged state of acute arousal. It is characterized by persistent baseline shifts, reduced regulatory stability over time, and disrupted acute stress reactivity, with responses that may be blunted, exaggerated, or slow to recover. For AI-based detection systems, this implies that chronic stress should be inferred from sustained deviations from normative baseline states, altered temporal variability, and maladaptive reactivity trajectories, rather than from short-lived stress-related increases in physiological signals alone. This biological view supports computational models that integrate longitudinal and multimodal physiological data in order to capture enduring stress-related dysregulation.

### 3.3. Labeling Strategies in Chronic Stress Research

In AI-based physiological modeling, the validity and interpretability of predictive systems depend strongly on how supervisory labels are defined. Unlike acute stress, which can be experimentally induced and localized in time, chronic stress is a prolonged and multidimensional construct that cannot be directly observed. Ground truth labels are therefore usually derived from validated psychometric instruments, biological markers, or documented long-term exposure contexts, which frequently serve as proxies for chronic stress rather than strict clinical diagnoses.

The PSS is the most widely used instrument for labeling sustained stress exposure in physiological and engineering studies [29,30,31,32,33,34,35,36,37,38,39,40,41,42,43,44]. Originally developed by Cohen et al. [7], the PSS measures the extent to which life situations are perceived as stressful over the previous month. The scale has shown good internal consistency, construct validity, and applicability across diverse populations [7,45]. In computational studies, PSS scores are commonly used either as continuous targets in regression models [31] or to divide participants into high- and low-stress groups for supervised learning [36]. Because its recall window covers approximately one month, the PSS offers a practical, although subjective, proxy for sustained stress exposure in AI-based stress detection.

The TICS was developed specifically to assess longer-term stress exposure across domains such as work overload, chronic worrying, social tension, and lack of social recognition [8]. In contrast to the PSS, the TICS generally assesses experiences over a three-month period, which more closely aligns with theoretical definitions of chronic stress based on sustained environmental demands and accumulated allostatic load. TICS-based labels have been used in studies examining the relationship between prolonged stress exposure and neurophysiological or autonomic markers [46], especially when domain-specific stressors are of interest.

The Depression Anxiety Stress Scales (DASS), particularly the DASS-21 and DASS-42 stress subscales, have also been used in physiological modeling studies as indicators of sustained stress-related symptoms. The DASS-Stress subscale measures persistent tension, irritability, and difficulty relaxing over the previous week [47]. Strong internal consistency and factorial validity have been reported in both clinical and nonclinical populations [48,49,50]. Although its recall window is shorter than that of the PSS or TICS, the DASS-Stress measure reflects ongoing physiological hyperarousal rather than immediate stress reactivity. For this reason, it has often been adopted in biomedical signal processing studies as a marker of subacute or sustained stress burden.

Furthermore, because chronic stress frequently manifests in occupational settings, domain-specific measures like the Maslach Burnout Inventory—General Survey (MBI-GS) and the Beck Depression Inventory (BDI) have been used to evaluate burnout as a proxy for chronic stress [51]. Other multimodal and cardiovascular frameworks have successfully utilized the Stress Response Inventory (SRI) [52,53] and the Connor-Davidson Resilience Scale (CD-RISC) [34] to contextualize physiological reactivity and sustained stress responses. By broadening the scope of these psychometric tools, researchers can better capture the multifaceted nature of allostatic load.

In addition to questionnaire-based measures, biological markers have been incorporated to provide more objective indicators of prolonged stress exposure. HCC has emerged as a retrospective biomarker that reflects integrated HPA-axis activity over periods of weeks to months [10,11]. Since scalp hair grows at approximately 1 cm per month, segmented hair analysis can be used to estimate cumulative cortisol secretion over extended periods [10]. In chronic stress studies, HCC has often been combined with psychometric measures to provide biologically grounded reference labels and to strengthen ecological validity [54,55].

Ground truth has also been derived from prolonged naturalistic exposure paradigms, such as extended academic examination periods or sustained occupational workload [56,57], which are utilized as related proxy constructs for modeling sustained stress. In such studies, physiological signals are recorded during documented periods of prolonged environmental demand, and stress status is inferred from contextual exposure rather than from self-report alone. These designs offer ecologically relevant supervisory information for modeling sustained stress states, although environmental exposure and subjective stress perception may not always coincide.

Across physiological sensing studies, the labeling framework selected for a study directly affects how features are interpreted and how predictive models are trained and evaluated. For this reason, the labeling strategy, including the recall window, cut-off criteria, and any biological or contextual references, should be clearly stated as a core methodological element in AI-based chronic stress research. Because no universal gold standard exists for chronic stress assessment, transparency in label construction remains essential for reproducibility, comparability, and appropriate interpretation of model performance.

### 3.4. Physiological Inference Paradigms for Chronic Stress Detection

Chronic stress is inherently a time-dependent condition marked by sustained physiological adaptation rather than short-lived activation. As a result, physiological sensing and computational studies have largely converged on three complementary paradigms for chronic stress inference: resting baseline dysregulation, longitudinal monitoring of physiological dynamics, and reactivity-based inference. Although these paradigms differ in implementation, each reflects a distinct but related manifestation of accumulated allostatic load.

The first paradigm, resting baseline dysregulation, is based on the assumption that chronic stress produces stable alterations in intrinsic physiological regulation that remain detectable even in the absence of an acute challenge. Prolonged exposure to stress mediators contributes to allostatic load and leads to persistent recalibration of autonomic, endocrine, and neural systems [5,24]. At the neural level, chronic stress has been linked to functional alterations in prefrontal–limbic circuits involved in emotion regulation and stress adaptation [24,58]. Computational studies have successfully classified these shifts using resting EEG spectral power and functional connectivity [29,32,51]. At the autonomic level, chronic stress is consistently associated with reduced resting HRV and altered EDA, suggesting diminished parasympathetic modulation and impaired neurovisceral integration [27,28]. Research utilizing multimodal fusion (EEG, ECG, and EDA) has mapped these baseline deviations to PSS labels using SVM and Multilayer Perceptron (MLP) [30]. Within the endocrine domain, resting-state statistical analysis of cortisol has further validated this paradigm as a route for identifying sustained dysregulation [52]. In this paradigm, chronic stress is inferred from coordinated deviations across neural, autonomic, and endocrine systems, including reduced variability, altered regulatory tone, and diminished adaptive capacity. Therefore, resting-state analysis provides a biologically grounded route for identifying sustained stress-related dysregulation.

The second paradigm, longitudinal monitoring, extends this perspective across time. Since chronic stress reflects cumulative biological burden, its detection cannot rely solely on a single recording session. Instead, physiological signals need to be aggregated across days or weeks. Repeated or continuous monitoring with wearable sensors, ambulatory ECG, or portable neurophysiological devices enables detection of gradual changes in autonomic tone, progressive suppression of variability [59], and altered circadian organization [57,60]. This approach aligns closely with the concept of allostatic load, in which chronic stress is understood as the cumulative wear and tear resulting from repeated activation of stress response systems [5,6]. From a computational standpoint, longitudinal inference requires temporal aggregation of features, normalization across circadian phases, and adjustment for contextual variables such as sleep, physical activity, and time of day. Without such controls, changes in the recorded signals may reflect ordinary behavioral fluctuation rather than sustained physiological dysregulation.

The third paradigm, reactivity-based inference, identifies chronic stress through altered responses to standardized acute challenges. Evidence suggests that prolonged stress exposure may be associated with attenuated or blunted physiological reactivity, reflecting reduced flexibility of stress-regulatory systems [23,61,62]. Rather than showing exaggerated responses, chronically stressed individuals may exhibit diminished cortisol reactivity [23], altered cardiovascular responses [61], or reduced neural modulation [63] during cognitive or emotional challenge tasks. Such blunted reactivity is consistent with allostatic models, in which repeated activation leads to longer-term recalibration of stress response systems [5,6]. In computational studies, acute stressors are therefore used not as targets in themselves but as probes to reveal latent chronic dysregulation. Stimulus-locked changes from baseline are compared across individuals stratified using validated chronic stress labels. The objective is not to detect acute stress alone, but to identify maladaptive response patterns that indicate longer-term physiological burden.

Across all three paradigms, circadian rhythmicity must be considered carefully. Cortisol follows a strong diurnal pattern, with a morning peak and gradual decline across the day, and HRV indices also vary with time of day [20,64]. If the circadian phase is not controlled, stress-related interpretations may be confounded. Resting-state and longitudinal studies, therefore, require temporal alignment and normalization, while reactivity-based paradigms depend on consistent baseline conditions to ensure that stimulus-evoked changes are interpreted appropriately.

Overall, these three approaches provide complementary biological perspectives on chronic stress detection. Persistent regulatory shifts are emphasized by baseline analyses, cumulative changes over time are captured by longitudinal monitoring, and adaptive flexibility under controlled perturbations is assessed by reactivity-based methods. A clear understanding of these inferential pathways is essential for designing AI models that can distinguish chronic stress from transient emotional fluctuations or isolated laboratory-induced stress responses.

**Figure 4:**
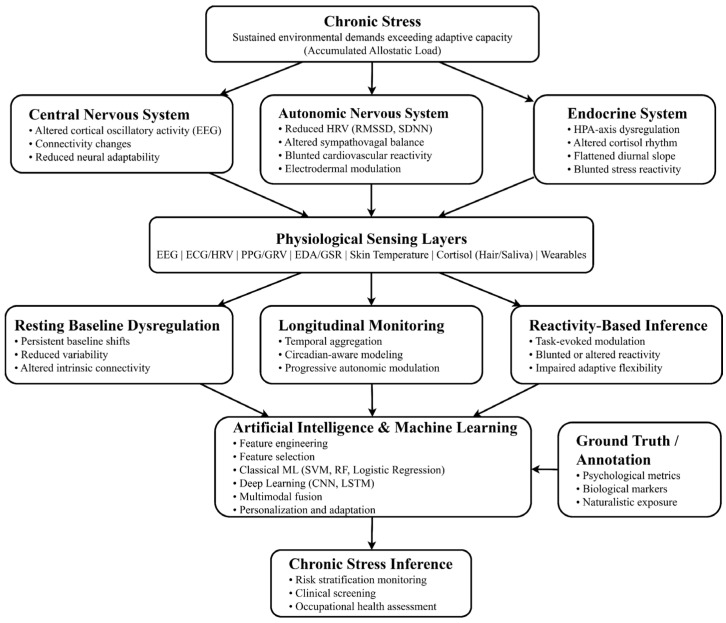
Conceptual framework of AI-based chronic stress detection using physiological sensing.

The multilevel relationship among chronic stress biology, physiological sensing modalities, inferential paradigms, and AI modeling is summarized in Figure 4. In that framework, central, autonomic, and endocrine dysregulation are linked to three main computational strategies: resting baseline analysis, longitudinal monitoring, and reactivity-based modeling, all anchored to their corresponding ground-truth and annotation schemes.

## 4. Physiological Sensing Modalities for Chronic Stress Detection

This section examines the various physiological sensing modalities used for chronic stress inference. Specifically, signals derived from the central nervous system (CNS), cardiovascular indices, electrodermal and peripheral autonomic measures, and biochemical markers are evaluated. Furthermore, frameworks relying on multimodal integration to capture complementary physiological pathways are discussed. Although a wide range of physiological modalities has been explored in the reviewed literature, cross-sectional resting-state recordings have been relied upon most heavily, whereas longitudinal or reactivity-oriented designs have been employed less frequently.

### 4.1. Central Nervous System Signals

Signals derived from the central nervous system (CNS) provide direct access to neural processes involved in stress perception and regulation. A range of neuroimaging modalities, including EEG, Magnetic Resonance Imaging (MRI), functional MRI (fMRI), Near-infrared Spectroscopy (NIRS), functional NIRS (fNIRS), and Magnetoencephalography (MEG), have been used to investigate stress-related brain activity. However, in the literature reviewed here, EEG was the only CNS modality consistently incorporated into computational frameworks for chronic stress detection. Although important mechanistic insights have been provided by fMRI, MRI, fNIRS, and MEG studies, such work has often been limited to group-level statistical comparisons or descriptive activation analyses rather than automated classification or scalable inference. By contrast, EEG has been more readily integrated into ML pipelines because of its high temporal resolution, portability, and relative suitability for wearable and repeated-measure settings.

#### 4.1.1. Resting-State EEG Markers

**Figure 5:**
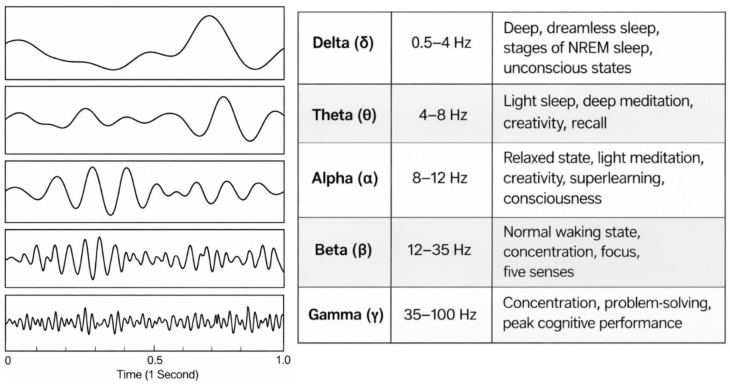
EEG spectral bands and their associated mental states are commonly utilized as features in stress detection frameworks.

Resting-state EEG has been widely used in supervised frameworks for detecting chronic or long-term stress. Across studies, spectral band power [65], as shown in Figure 5, has been the most frequently extracted feature class, with particular attention given to beta- and gamma-band activity. Elevated beta power has been associated with higher perceived stress [52], and correlation-based feature selection has identified low-beta, high-beta, and low-gamma components as being strongly related to stress indices [37]. Additional discriminative value has been reported for features of hemispheric asymmetry. In one long-term stress classification study, frontal and temporal alpha asymmetry was identified as a significant biomarker within an SVM-based framework [39]. In reduced-channel wearable EEG systems, theta-band features have also been shown to provide strong predictive utility and to be associated with high accuracy in binary stress discrimination tasks [30,37].

Although band-power features have dominated this literature, reliance on linear spectral measures alone may restrict model sensitivity. Chronic stress-related conditions, such as burnout, which may arise from prolonged occupational stress exposure, have been associated with altered resting-state functional connectivity, particularly reduced frontal alpha-band connectivity relative to controls [51]. It has therefore been suggested that connectivity-based features, including coherence and phase synchronization, may offer richer representations of stress-related network-level recalibration. Nonlinear EEG descriptors have also been reported to be informative. For example, fractal dimension and other higher-order dynamical features derived from resting EEG were shown to distinguish individuals with elevated chronic stress scores [29]. Such measures may better capture reduced neural adaptability under accumulated allostatic load. From a modeling perspective, improved feature-space separability can be achieved by incorporating connectivity and nonlinear descriptors, even without increasing the channel count.

#### 4.1.2. Longitudinal EEG Monitoring

In contrast to wearable cardiovascular approaches, longitudinal EEG monitoring for chronic stress has remained largely underexplored in the reviewed literature. Most EEG-based chronic stress studies have relied on single-session recordings obtained during resting or task-based conditions, with labels derived from validated psychometric scales, such as the PSS. Although these designs have enabled discrimination between participants with higher and lower perceived stress, temporal stability, and progressive neural adaptation have not been directly examined across extended periods of observation.

Accordingly, the available evidence supports the use of EEG spectral and asymmetry features as cross-sectional markers of perceived chronic stress. However, systematic longitudinal EEG tracking across weeks or months has not yet been established in the reviewed studies. Relative to wearable cardiovascular monitoring, prolonged neural monitoring of chronic stress remains a clear methodological gap.

#### 4.1.3. EEG Reactivity Paradigms

EEG reactivity paradigms have been used to assess chronic or perceived stress by examining task-evoked neural responses rather than resting activity alone. In such studies, participants were stratified using validated stress measures, such as the PSS or related inventories, and were then exposed to cognitive or affective challenges, including Stroop tasks and mental workload paradigms, while EEG was recorded [37,38,42,66]. Spectral features obtained during task engagement were subsequently compared across stress groups.

In Stroop-based designs, task-evoked EEG activity has shown meaningful discriminative value for chronic stress classification, with alpha-, beta-, and gamma-band modulation contributing to model performance [66]. Comparable findings were reported in wearable EEG headband studies, in which band power and asymmetry features varied with perceived stress and supported successful SVM-based classification [37]. In context-aware EEG frameworks, differential entropy (DE) features extracted during emotional transition states were also found to vary with stress level, suggesting that task-dependent neural regulation may be altered under higher perceived stress [38].

Across these studies, chronic stress was not primarily characterized by exaggerated neural activation. Rather, it was reflected in altered alpha suppression and modulation of beta and gamma activity during cognitive engagement. These findings support the view that chronic stress may manifest as reduced neural flexibility under controlled perturbation and reflect impaired adaptive regulation after prolonged stress exposure.

### 4.2. Cardiovascular Signals

Cardiovascular signals, especially HRV, ECG, and PPG-derived indices, are among the most extensively used physiological domains for chronic stress inference. Across resting, repeated-measures, and reactivity-based paradigms, autonomic markers have consistently served as discriminative features for ML-based stress classification.

#### 4.2.1. Resting Autonomic Imbalance

Resting HRV and ECG-derived features have been used in several studies to distinguish individuals with elevated chronic stress scores. In one study, HRV features extracted from ECG signals were used to classify participants by self-reported stress level, and reliable discrimination was achieved using time- and frequency-domain measures in supervised models [53]. Likewise, in multimodal biosignal frameworks, resting HRV indices derived from ECG were reported to contribute substantially to chronic stress classification [52].

**Figure 6:**
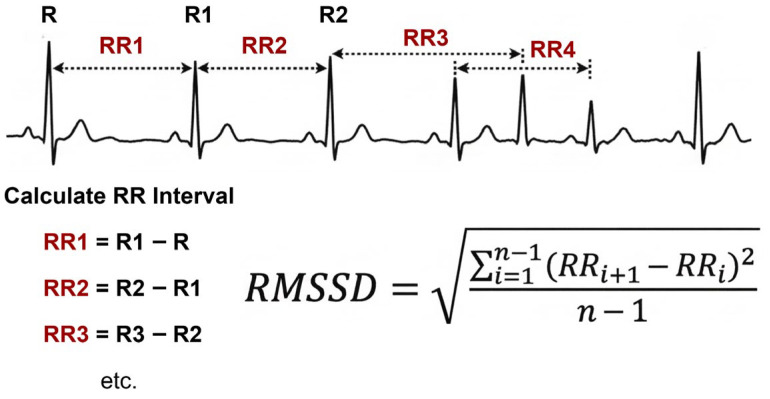
Visual representation of the calculation for the RMSSD, a primary time-domain feature used to quantify HRV from the RR intervals in an ECG signal.

The most commonly extracted features included time-domain measures such as RMSSD [67] (illustrated in Figure 6) and SDNN, together with frequency-domain features such as low-frequency (LF) and high-frequency (HF) power. Although the reported direction of change was not entirely consistent across studies, reduced variability and altered sympathovagal balance were frequently associated with higher stress levels [52,53]. Taken together, these findings suggest that chronic stress is accompanied by a measurable autonomic imbalance that can be detected under resting conditions. From a computational standpoint, resting HRV measures remain attractive because they are physiologically interpretable and readily incorporated into standard ML classifiers.

#### 4.2.2. Longitudinal Wearable Monitoring

Wearable cardiovascular systems extend stress inference beyond single-session assessment by enabling HRV- or PPG-derived features to be extracted over repeated monitoring windows. In one two-stage anomaly filtering framework for continuous stress monitoring, PPG-derived HRV features were aggregated to generate robust stress predictions under real-world conditions [31]. A similar two-stage architecture was also implemented in a consumer-grade wearable study to detect chronic stress using longitudinal physiological streams [43].

In other work, PPG-derived heart-rate features were combined with contextual or behavioral variables, including sleep and activity patterns, to improve classification performance during daily life monitoring [40]. In older adult cohorts, PPG-based models were also shown to retain their informative value under ambulatory conditions, with heart rate dynamics and variability indices contributing to stress inference [68]. Although progressive autonomic drift was not always modeled explicitly, these studies indicate that greater robustness may be achieved by aggregating cardiovascular features across repeated sessions rather than analyzing isolated episodes. Overall, wearable and repeated-measures cardiovascular studies suggest that inference of chronic stress is strengthened by temporal aggregation of HRV-related features across naturalistic settings [31,40,43,68].

#### 4.2.3. Reactivity-Based Autonomic Inference

Reactivity-based cardiovascular paradigms have been used to assess chronic stress through autonomic responses elicited during controlled perturbation. In experimental designs involving cognitive load, cardiovascular responses, such as heart rate, pulse wave amplitude, and related variability measures were found to differ significantly between higher- and lower-stress groups [62]. In particular, attenuated physiological reactivity during task engagement was observed in participants with higher stress, suggesting blunted autonomic responsiveness under sustained stress exposure.

Related findings were reported in studies examining acute stress reactivity and recovery, in which HRV-derived features obtained during perturbation phases provided useful information for stress stratification [34]. Common task-evoked features included changes in RMSSD, modulation of the LF/HF balance, and heart rate elevation during cognitive demand. ML models trained on such task-induced cardiovascular measures reliably classified stress, indicating that autonomic modulation during challenge may provide information that complements resting-state assessment [34,62].

### 4.3. Electrodermal and Peripheral Autonomic Signals

EDA, galvanic skin response (GSR), and related peripheral autonomic measures, including skin temperature, are commonly treated as markers of sympathetic nervous system activity and are frequently included in stress detection frameworks. Within the reviewed studies, these signals were rarely used as standalone predictors of chronic stress. More commonly, they were incorporated into multimodal systems together with cardiovascular and motion-related features.

Under resting conditions, tonic skin conductance and statistical descriptors of electrodermal dynamics were found to provide information complementary to HRV-based autonomic measures [30,69]. Although resting EDA alone did not consistently separate chronic stress groups, classification performance was often improved when EDA was fused with cardiovascular signals. Peripheral skin temperature, which may reflect vasoconstrictive changes related to sympathetic activation, was also included in some wearable systems to support contextual interpretation of autonomic state [69].

In repeated-measure and wearable monitoring studies, electrodermal signals were extracted over predefined time windows to support stress inference in naturalistic environments [33,41]. Typically, tonic and phasic conductance components, response amplitudes, and temporal variability measures were computed and aggregated over monitoring intervals. When combined with PPG-derived heart rate and activity features, EDA was found to improve robustness compared with single-episode measurements. Although progressive sympathetic drift was not directly demonstrated in these studies, stable stress recognition in ambulatory settings was supported by aggregation of electrodermal dynamics across sessions [41].

More direct evidence for the role of electrodermal signals was observed in reactivity-based paradigms. During cognitive challenge tasks, altered GSRs were reported in individuals with higher chronic stress scores relative to lower-stress participants [62]. Higher-stress groups showed attenuated sympathetic reactivity under cognitive load, consistent with blunted autonomic responsiveness under sustained stress exposure. Reliable classification performance was achieved when ML models were trained on task-evoked electrodermal features, often in combination with cardiovascular signals [62]. In these settings, relevant markers included changes in skin conductance amplitude and response frequency during stress-inducing tasks.

Overall, electrodermal and related peripheral autonomic signals appear to contribute to chronic stress inference mainly through sympathetic response patterns observed under controlled challenge and through their aggregation in wearable multimodal frameworks. Their discriminative value appears to be strongest when they are integrated with cardiovascular features rather than applied in isolation.

### 4.4. Biochemical Markers

Compared with neurophysiological and cardiovascular signals, biochemical markers have been incorporated only infrequently into AI-based frameworks for chronic stress detection. Among the reviewed studies, cortisol was the primary endocrine variable included in the computational analyses.

In one multimodal framework combining EEG, ECG, and salivary cortisol, cortisol concentration was used as a physiological reference for stress levels and was interpreted alongside neural and autonomic features [52]. Elevated salivary cortisol was associated with reduced HRV, as indexed by SDNN, and with increased frontal high-beta EEG activity, suggesting convergence across endocrine activation, autonomic regulation, and cortical oscillatory dynamics [52]. Although cortisol was not always used as a standalone predictor, its inclusion provided biological support for stress stratification and improved interpretation of multimodal feature patterns.

Beyond this type of integration, biochemical markers have not yet been systematically incorporated into ML pipelines for chronic stress detection. None of the reviewed studies implemented long-term endocrine-tracking approaches, such as hair cortisol concentration analysis or diurnal cortisol slope modeling, within computational classification frameworks. At present, biochemical measures appear to function mainly as complementary or validation-oriented variables rather than as continuous predictive inputs.

### 4.5. Multimodal Integration

Multimodal systems, as ML frameworks that integrate two or more physiological modalities, were widely represented in the reviewed literature. The purpose of such systems is to capture complementary aspects of stress physiology by combining central, autonomic, and peripheral signals within a unified computational framework.

In several cross-sectional studies, CNS and autonomic measures were fused at the feature level. For example, one multimodal framework integrated EEG, ECG-derived HRV, and salivary cortisol to classify chronic stress, thereby linking cortical oscillatory activity, autonomic variability, and endocrine activation within a single model [52]. In that design, spectral EEG features, HRV indices, such as SDNN, and cortisol concentrations were combined prior to supervised classification, illustrating the value of integrating neural and peripheral stress pathways.

Wearable multimodal systems frequently combine cardiovascular and electrodermal features. In mobile and consumer-grade implementations, PPG-derived HRV, EDA, motion signals, and, in some cases, skin temperature, were integrated to support stress recognition under ambulatory conditions [41]. In these architectures, modality-specific features, including RMSSD from PPG, tonic and phasic EDA components, and activity-related measures, were concatenated before classification.

The complementary value of multimodal integration was also illustrated in task-based paradigms. In cognitive challenge studies, cardiovascular responses, electrodermal activity, respiratory measures, and pupil diameter were fused to distinguish chronic stress groups [62]. In these settings, improved discrimination was obtained when sympathetic, cardiovascular, and peripheral vascular signals were modeled jointly rather than separately. Participants with higher chronic stress frequently exhibited attenuated autonomic reactivity across multiple physiological domains, further supporting the use of integrated modeling [62].

Across the reviewed literature, feature-level concatenation was the most common fusion strategy. Modality-specific features were usually merged before training classifiers, such as SVM, Random Forests (RF), or MLP. Decision-level fusion was reported less often. Overall, multimodal integration was generally associated with better classification performance than unimodal modeling, particularly when EEG was combined with autonomic measures, such as HRV and EDA or when cardiovascular and electrodermal features were aggregated in wearable systems.

## 5. AI Methods in Chronic Stress Detection

**Figure 7:**
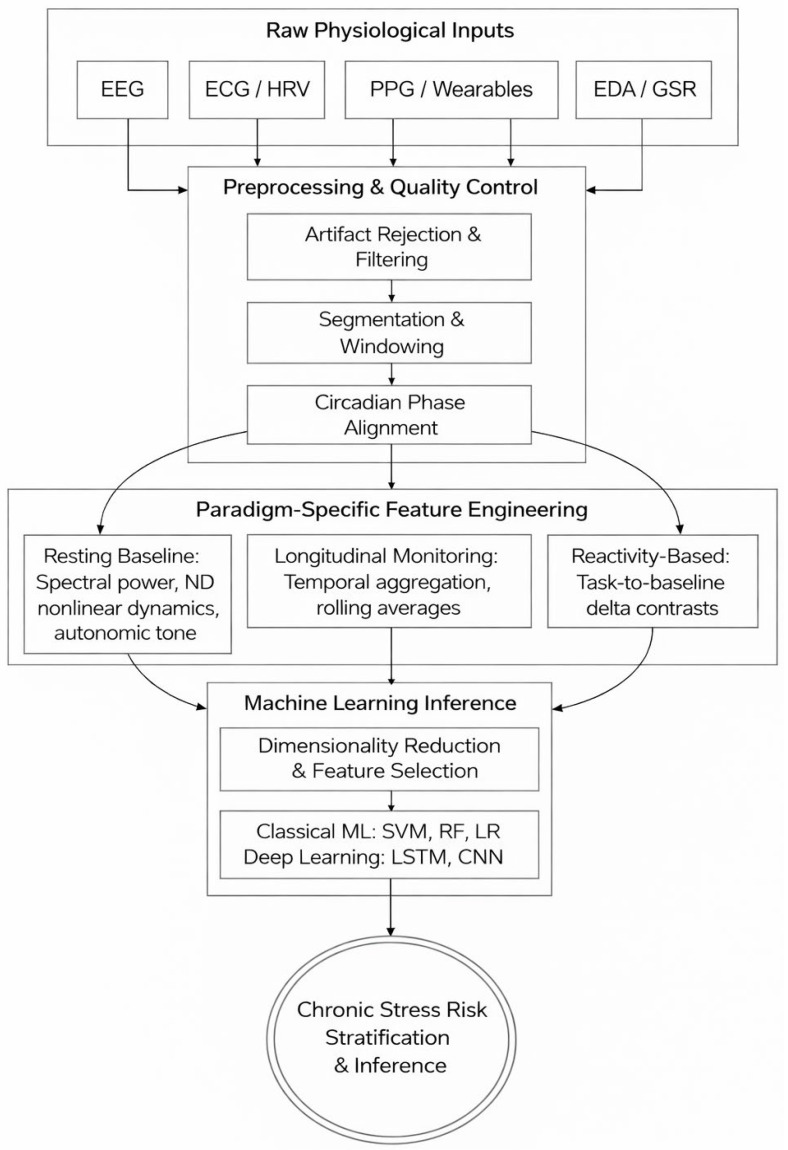
Specialized AI and ML pipeline for chronic stress detection.

Although physiological sensing provides candidate biomarkers of chronic stress, the utility of these signals depends on whether they can be converted into inference systems that are reliable, generalizable, and suitable for deployment. Within the reviewed literature, AI and ML are utilized to manage high-dimensional physiological features and perform predictions despite substantial inter-individual variability. However, the application of AI to chronic stress extends beyond generic classification; it requires specialized computational pipelines tailored to capture accumulated allostatic load. Figure 7 illustrates this specialized computational flow, mapping the progression from raw signal acquisition through targeted preprocessing, paradigm-specific feature engineering, and final ML inference. As shown in the pipeline, the specialization for chronic stress generally does not lie in the core classification algorithms themselves, but rather in how the data preprocessing and feature engineering architectures are structured to handle time-dependent physiological dysregulation.

### 5.1. Signal Quality Control and Preprocessing

As depicted in the second layer of the computational pipeline (Figure 7), raw physiological signals, such as PPG, EDA, and EEG, are highly vulnerable to motion artifacts, contact instability, and other sources of noise in wearable and ambulatory settings. Consequently, robust preprocessing is introduced before any stress inference can occur. Beyond standard artifact rejection and filtering, chronic stress pipelines often require specialized segmentation and circadian phase alignment. Because autonomic baselines fluctuate naturally throughout the day, normalizing signals to account for time-of-day effects is frequently necessary to isolate true allostatic dysregulation from ordinary diurnal variation.

In several wearable PPG-based studies, two-stage frameworks were adopted in which signal-quality assessment was separated from stress prediction to improve robustness [31,43]. Comparable emphasis on preprocessing and segmentation quality has also been reported in wearable stress resources, such as WESAD, where reliable modeling depends heavily on careful preparation of the signal stream [70]. Thus, AI and automated filtering are used not only for classification or regression but also for the stabilization of noisy biosignals in real-world environments.

### 5.2. Feature Representation and Paradigm-Specific Engineering

Most chronic stress detection systems are built upon physiologically motivated, handcrafted features. Because classical ML algorithms typically evaluate segmented data windows as independent observations, the specialization for chronic stress primarily occurs during the feature engineering stage (as outlined in the third layer of Figure 7). The extracted representations must align directly with the underlying inferential paradigm to successfully capture allostatic load.

For resting baseline dysregulation, models rely on engineered features that capture persistent regulatory shifts, such as nonlinear descriptors of neural adaptability, resting autonomic tone, and spectral band power [29]. For longitudinal monitoring pipelines, extracting static features is insufficient; systems must compute temporal aggregation, rolling averages, and progressive modulation trends from raw sequences to model the gradual accumulation of stress over time [31,43]. Finally, in reactivity-based paradigms, raw signal amplitudes are rarely fed directly into classifiers. Instead, specialized pipelines compute task-to-baseline delta contrasts to isolate blunted or altered stress-related modulation during challenge conditions [39,52,66,71].

While DL methods, such as Long Short-Term Memory (LSTM) networks, have been introduced to automatically learn temporal dependencies directly from raw sequences [72,73], their application in chronic stress cohorts remains limited by small sample sizes. Consequently, paradigm-specific feature engineering followed by classical ML classification remains the most robust and dominant computational approach in this field.

### 5.3. Feature Selection and Dimensionality Reduction

Managing high-dimensional physiological data is critical in chronic stress research, particularly for EEG-based features, given the typically small sizes of available cohorts. For instance, some analytical pipelines extract hundreds or even thousands of candidate EEG features [29]. Without applying dimensionality reduction, the combination of vast feature spaces and small sample sizes frequently leads to overfitting and artificially inflated performance metrics. To mitigate this, techniques such as sequential backward selection have been effectively used to eliminate redundant variables and enhance model generalization [71].

Optimizing feature subsets not only improves statistical stability and model interpretability, but also significantly reduces computational overhead. These benefits are especially crucial for wearable and reduced-channel EEG systems, which demand high operational efficiency. While several foundational and laboratory-based studies evaluate small cohorts of fewer than 30 participants [29,71], there is a growing trend toward larger datasets, particularly in wearable and longitudinal research. Recent studies have successfully reached sample sizes of *n* = 104 and *n* = 131. Despite this increase in participant numbers, managing high-dimensional physiological features remains a fundamental requirement to prevent overfitting, as the number of extracted features often still exceeds the available sample size.

### 5.4. Classical Machine Learning

Classical ML remains the predominant modeling strategy in studies of chronic and perceived stress detection. This preference appears to be largely driven by two practical conditions: most pipelines rely on engineered physiological features, and most available datasets are relatively small. Among the foundational methods, SVM and Support Vector Regression (SVR) are the most frequently used [29,30,31,32,33,36,37,39,43,66,68,71]. Other traditional baselines are also consistently reported across both single-modality and multimodal frameworks; these include Naïve Bayes (NB) [30,36,37,39,62], k-Nearest Neighbors (k-NN) [29,39,42], Linear Discriminant Analysis (LDA) [71], and MLP[30,31,36,37,39,62,68]. Logistic regression (LR) has also been frequently utilized, particularly in time-dependent HRV studies and multimodal designs to separate higher- and lower-stress groups [39,53,62,66,68].

To handle complex physiological data and nonlinear feature interactions more robustly, researchers often progress to ensemble methods. Tree-based ensembles, particularly RF, have been widely adopted across various designs, including Stroop-based EEG classification, multimodal wearable frameworks, and consumer-grade wearable systems [31,42,43,62,66,68]. Building on this, advanced ensemble architectures, such as LightGBM [40] and ExtraTree [42,68], have been effectively implemented to maintain high performance under noisy wearable conditions.

Beyond supervised learning, unsupervised approaches have been explored, such as applying k-means clustering in mobile physiological response frameworks to identify stress-related groupings [69]. Taken together, the reviewed studies show that classical ML remains attractive because it is feasible under limited-data conditions and usually offers a degree of interpretability. However, these models generally treat segmented windows as independent observations. As a result, temporal dependencies, such as gradual longitudinal modulation or altered reactivity dynamics, are not learned directly by the model and instead must be introduced through feature engineering and temporal aggregation [31,43].

### 5.5. Deep Learning Approaches

In chronic stress-specific research, DL is gradually emerging, although it has not yet been as widely adopted as classical ML. Neural-network-based modeling has been explored within a time-slot framework, in which ambulatory ECG and triaxial acceleration signals were used to recognize chronic stress [35]. These findings indicate that DL is feasible for longitudinal wearable monitoring.

Furthermore, while convolutional neural networks (CNNs) have traditionally been applied mostly in the broader literature on acute stress and affective computing to automatically learn spatial–spectral patterns, recent frameworks have successfully adapted them specifically for chronic stress. For instance, a CNN-based context-aware model has been utilized to classify perceived chronic stress using differential entropy features derived from EEG during a reactivity paradigm [74]. Recurrent neural networks, especially LSTM architectures, have likewise been explored for wearable stress detection using signals, such as PPG and EDA [72,73]. These approaches are appealing because they can model temporal dependencies more directly than conventional classifiers.

Despite these methodological advances, the majority of published chronic stress studies continue to rely on engineered physiological features combined with conventional ML classifiers. While DL models offer powerful feature-learning capabilities, their limited adoption in chronic stress research likely reflects the constraints of small dataset sizes, session-level labeling, and the current lack of standardized longitudinal benchmarks.

### 5.6. Modeling Strategies and Generalization

Most chronic stress detection systems are formulated as binary classification problems, where participants are divided into high- and low-stress groups based on questionnaire thresholds [29,30,37,66]. The Perceived Stress Scale (PSS) is the most frequent instrument used for this labeling, appearing in studies ranging from single-channel EEG [32] to multimodal smartwatch frameworks [40]. Other validated tools, such as the Stress Response Inventory (SRI) [52,53] and the State-Trait Anxiety Inventory (STAI) [62], are also employed. While binary labels are convenient for screening, they can obscure intermediate physiological states. To address this, some studies have implemented continuous stress estimation using SVR to predict longitudinal scores [31,33]. These regression approaches preserve the continuous nature of stress-related outcomes, but they require more careful calibration to remain stable across different environments.

A critical distinction in the current literature is the widespread reliance on subject-dependent validation rather than subject-independent validation. In subject-dependent modeling, common in EEG-based studies, training and evaluation data are often pooled from the same cohort using standard k-fold cross-validation or simple train/test splits [32,36,37,66]. Because baseline physiological signals are highly individualized, models trained this way frequently learn subject-specific traits rather than generalizable stress signatures, leading to heavily overestimated performance. For wearable health AI, subject-independent validation is absolutely crucial. Fortunately, several studies have successfully implemented subject-independent strategies. While Leave-One-Subject-Out (LOSO) cross-validation is commonly used to ensure the model generalizes to unseen individuals [29,30,41], other frameworks have adopted participant-level splits [38], epoch-level isolation (1 participant = 1 epoch) [40], individual-level aggregation [43], and subject-wise 10-fold cross-validation [62]. This approach is especially important for heterogeneous populations, such as older adult cohorts, where individual differences in physiological baselines can lower the performance of models that are not generalized [68]. While some wearable chronic stress studies have placed greater emphasis on cross-subject robustness [31,43], true external validation remains rare. Until the field consistently adopts subject-independent testing protocols, the clinical and commercial viability of these AI models cannot be confidently established.

Personalization remains a significant limitation in current systems. Chronic stress is a deeply individualized and time-dependent process, yet adaptive mechanisms like transfer learning, domain adaptation, and online updating are rarely integrated into AI pipelines. Future research should focus on hybrid architectures that combine population-level generalizability with subject-specific tuning. This strategy is essential for moving from laboratory-based stress detection to real-world monitoring systems that can accurately track long-term allostatic dysregulation.

### 5.7. Edge AI and Real-World Deployment

For wearable chronic stress monitoring, computational efficiency, energy constraints, and robustness to motion-related disturbance are all major practical considerations. Several wearable frameworks in the reviewed literature have therefore adopted multi-stage designs in which signal-quality assessment or anomaly filtering is performed separately from the final inference stage [31,43]. In contrast to laboratory EEG systems, wearable implementations have generally placed greater emphasis on preprocessing procedures, such as normalization, contextual filtering, and temporal feature aggregation to reduce the effects of motion artifacts and circadian variability [40,41]. These measures are necessary if stress monitoring is to be performed outside controlled laboratory conditions.

Even so, most chronic stress systems remain dependent on offline processing and feature engineering rather than on fully embedded, end-to-end on-device inference. Real-world deployment is therefore still limited by several unresolved issues, including small training datasets, weak longitudinal validation over extended monitoring periods, the lack of standardized chronic stress benchmarks, and inconsistent subject-independent evaluation protocols. As a result, although strong experimental results have been reported, ecological robustness and translational scalability remain insufficiently established for routine chronic stress monitoring applications [31,41,43].

Table 2 provides a structured summary of the reviewed studies, including the main physiological modalities, inferential paradigms (resting, longitudinal, and reactivity-based), feature categories, and AI models used for chronic stress detection. Because the reviewed studies utilize highly heterogeneous datasets, labeling strategies, and validation protocols, direct quantitative comparisons of classification accuracies are intentionally omitted to avoid misleading performance rankings. Instead, this summary serves to map the current methodological landscape and highlight prevailing analytical trends.

## 6. Methodological Challenges in Chronic Stress AI Research

Despite growing interest in physiological sensing and ML for chronic stress detection, several methodological limitations still affect the validity, interpretability, and generalizability of current systems. These limitations arise at multiple levels, including label definition, study design, temporal control, between-subject variability, and dataset size.

### 6.1. Labeling Ambiguity

One of the main difficulties in chronic stress research lies in how chronic stress is defined operationally for model development. In most reviewed studies, labels are derived from self-report instruments, most commonly the PSS [29,37,66]. Although such instruments are validated for measuring perceived stress over recent weeks, they are often paired with short physiological recordings, such as brief resting EEG or HRV segments. As a result, a mismatch may arise between a trait-level psychological construct and a momentary physiological measurement.

A further issue is that continuous questionnaire scores are frequently converted into binary categories, such as high versus low stress, to simplify supervised classification [30,37,66]. While this strategy is practical, part of the underlying variation in stress severity may be lost. Similar ambiguity is present in task-based paradigms, where physiological responses to acute challenges are interpreted using trait-level stress labels [66]. Under these conditions, it is not always clear whether the detected patterns reflect altered stress reactivity, persistent baseline dysregulation, or both.

Furthermore, it is critical to acknowledge the boundary between clinically diagnosed chronic stress and related psychological constructs. Because large-scale physiological datasets with true clinical chronic stress labels are exceedingly rare, many studies rely on proxies, such as high perceived stress scores, occupational workload assessments, or burnout inventory scales. While these constructs are highly valuable for modeling different facets of accumulated allostatic load, they are not strictly equivalent to a clinical medical diagnosis. This reliance on proxy labels further compounds labeling ambiguity, making it difficult to precisely evaluate the clinical validity of current AI frameworks.

Overall, questionnaire-based labeling remains convenient and widely used, but its combination with short-duration physiological recordings introduces uncertainty in the interpretation of model outputs. It therefore remains difficult to determine whether the identified signatures reflect enduring chronic stress or temporary context-dependent activation.

### 6.2. Limited Longitudinal Validation

Because chronic stress is inherently time-dependent, its assessment would ideally require repeated measurements across extended periods. However, most reviewed studies rely on cross-sectional or single-session designs. In many EEG-based frameworks, physiological data are collected during one laboratory session and then used for classification without follow-up assessment [29,37,66]. Although such designs allow controlled comparison between groups, temporal stability is not directly evaluated.

Wearable monitoring studies move somewhat closer to longitudinal assessment. Multi-stage PPG-based systems [31] and consumer wearable frameworks [43] have enabled continuous or ambulatory acquisition under real-world conditions. Even so, the observation windows remain relatively limited and do not usually extend across the weeks or months that would better reflect cumulative allostatic burden. In addition, performance is commonly assessed via cross-validation on the same dataset, whereas stability over longer time intervals is rarely examined.

For this reason, current systems may demonstrate classification performance under controlled or short-term monitoring conditions, but evidence for true longitudinal validity remains limited. Without repeated observations over longer durations, it cannot be confidently determined whether the detected physiological patterns represent stable chronic dysregulation or only short-term fluctuations.

### 6.3. Circadian and Context Confounding

Physiological markers commonly used in stress research, including HRV, electrodermal activity, and cortical oscillatory measures, are strongly influenced by circadian rhythms and contextual factors such as posture, physical activity, and sleep–wake state. In laboratory EEG-based studies, recordings are usually obtained under controlled conditions [37,66]. Although this reduces environmental variability, it also reduces ecological validity. In contrast, wearable systems collect data in ambulatory settings, where motion artifacts and day-to-day contextual variation introduce additional complexity [31].

To improve robustness, preprocessing procedures such as artifact filtering, normalization, and feature aggregation across time windows are often applied in wearable frameworks [43]. These steps help stabilize the signal under real-world conditions. However, circadian phase and time-of-day effects are not modeled consistently across studies. If temporal factors are not controlled explicitly, natural diurnal changes in autonomic and neural signals may influence the features used for stress inference.

As a result, it remains difficult in many cases to separate physiological changes related to chronic stress from those associated with ordinary daily variation. Greater use of time-aware modeling and contextual metadata would likely improve both interpretability and model stability.

### 6.4. Inter-Individual Variability

Substantial variability exists across individuals in baseline autonomic activity, neural oscillatory patterns, and physiological reactivity. Measures commonly used for chronic stress detection, such as HRV indices and EEG spectral power, are also affected by age, sex, fitness, sleep quality, and lifestyle factors that are not specific to stress status. In most reviewed studies, models are trained on pooled participant data without explicit personalization mechanisms [29,36,66].

Wearable frameworks often include normalization and preprocessing steps to reduce variability under ambulatory conditions [31,43]. However, more explicit adaptive strategies, such as subject-specific calibration, transfer learning, or online model updating, have not been incorporated systematically into the reviewed chronic stress systems. Consequently, some classifiers may capture between-subject physiological differences rather than stress-related signatures alone.

This issue may become even more pronounced in multimodal systems, where heterogeneous features are combined within a single model. Without structured personalization or stratified modeling, inter-individual heterogeneity remains a persistent source of bias and a major obstacle to robust inference of chronic stress.

### 6.5. Small Sample Sizes

Limited sample size remains a recurring weakness in chronic stress AI research. Several EEG-based studies rely on relatively small cohorts [29,37,66], increasing the risk of overfitting, especially when high-dimensional spectral or nonlinear features are used. Although cross-validation is commonly reported, validation on small samples may still yield overly optimistic performance estimates.

Similar limitations are present in multimodal and wearable studies, where participant numbers are frequently restricted as well [30,31,36,69]. This reduces statistical power and weakens confidence in model generalization. The problem becomes more serious when feature spaces expand through multimodal fusion or when nonlinear classifiers are used. Furthermore, small sample sizes severely limit the ability to perform rigorous subject-independent validation; when cohorts are too small, withholding entirely unseen subjects for testing often leaves insufficient data for robust model training. Consequently, independent external validation is rarely reported in the reviewed literature.

## 7. Emerging Research Gaps and Future Directions

Despite continued progress in physiological sensing and AI, chronic stress detection remains methodologically fragmented and only partly aligned with real-world clinical or occupational use. Several gaps remain evident across the reviewed literature, particularly in dataset standardization, multimodal biological integration, model design, personalization, and clinical validation. Addressing these issues will be necessary if current experimental frameworks are to develop into scalable and clinically meaningful systems.

### 7.1. Standardized Chronic Stress Datasets and Benchmarking

In contrast to acute stress research, where public benchmark datasets are more readily available, chronic stress detection is still hindered by the lack of standardized, openly accessible datasets with consistent labeling procedures. Existing studies have generally been conducted on independent cohorts using different questionnaire thresholds, recording protocols, and experimental settings. As a result, direct comparison across studies remains difficult, and objective benchmarking of ML models is limited.

Future work should therefore be directed toward the development of multi-site and longitudinal cohort datasets that combine repeated physiological measurements with validated chronic stress instruments. Monitoring over longer periods, such as weeks or months, would allow temporal stability, predictive drift, and progression of physiological dysregulation to be examined more directly. Shared evaluation protocols, including subject-independent validation standards, would also be needed to enable fair comparison of competing models.

### 7.2. Multimodal Integration and Biological Grounding

Although multimodal physiological fusion has been reported in several studies, integration has typically been performed via simple feature concatenation prior to classification. More structured fusion strategies, including hierarchical, graph-based, or attention-guided architectures, have received relatively little attention in chronic stress-specific work. Given that chronic stress affects interacting neural, autonomic, and endocrine systems, stronger modeling of cross-modal relationships may improve robustness, interpretability, and biological relevance.

A further limitation is that endocrine pathways remain underrepresented in current computational pipelines. From a biological perspective, chronic stress is closely tied to dysregulation of the hypothalamic–pituitary–adrenal axis, yet markers such as cortisol are only rarely incorporated into AI-based frameworks. Most current systems rely mainly on autonomic or neurophysiological signals, and validation across neural, autonomic, and endocrine domains remains limited. Broader inclusion of biologically grounded multimodal data would likely strengthen the physiological interpretation of model outputs.

### 7.3. Hybrid Longitudinal and Reactivity Models

Most current studies emphasize either resting-state classification or task-based reactivity analysis, whereas few frameworks attempt to integrate both perspectives within a single modeling strategy. This separation is a limitation because chronic stress is expressed not only as baseline dysregulation but also as altered responsiveness to challenge. A more complete representation of chronic stress would therefore be expected from models that combine resting physiological markers, temporally aggregated monitoring data, and reactivity features obtained during controlled perturbation.

Such hybrid approaches may help bridge the mismatch between trait-level labeling and short-duration physiological measurement. They may also provide a more realistic account of chronic stress as a condition involving both persistent recalibration and impaired adaptive flexibility. For this reason, combined baseline-reactivity frameworks should be considered an important direction for future research.

### 7.4. Personalized and Adaptive AI Frameworks

Inter-individual variation in baseline physiological patterns remains a major obstacle to generalizable inference about chronic stress. Most existing systems have been trained on pooled population data, while structured personalization has rarely been incorporated. As a result, model performance may be reduced when physiological variability unrelated to stress differs substantially across individuals or populations.

Future systems would likely benefit from adaptive strategies, such as subject-specific calibration, transfer learning, domain adaptation, or incremental model updating. Hybrid frameworks that combine population-level inference with individualized adjustment may be especially useful in wearable or longitudinal settings, where physiological baselines differ substantially among users. Greater personalization may therefore improve both ecological validity and robustness in real-world deployment.

### 7.5. Explainable and Clinically Validated Systems

Although classification accuracy is frequently emphasized, explainability and clinical validation remain insufficiently developed. Without a clearer clinical evaluation, it remains uncertain whether AI-derived physiological markers provide diagnostic or prognostic value beyond established self-report instruments. Strong experimental performance alone is therefore not enough to justify practical adoption.

Future work should place greater emphasis on transparent modeling approaches in which predictions can be linked to physiologically interpretable features. Such interpretability will be important for clinician trust, responsible implementation, and meaningful use in occupational and mental health settings. It should also be examined whether physiological stress inference can predict clinically relevant outcomes, such as burnout progression, mental health decline, or cardiometabolic risk. For broader deployment, subject-independent validation, regulatory alignment, and cost-effectiveness will also need to be addressed more directly.

## 8. Conclusions

The reviewed literature shows that AI-based physiological sensing can help identify chronic stress-related dysregulation across neural, autonomic, and endocrine domains. However, because most current studies rely on related proxy constructs such as perceived stress, burnout, or task-based reactivity rather than clinical diagnoses, the ability to draw definitive conclusions about true chronic stress remains limited. Across the literature, EEG-derived neural markers, HRV-based autonomic indices, electrodermal signals, and wearable multimodal systems have demonstrated value in stratifying these related stress constructs. Most current systems still rely on classical ML, reflecting the small size and limited standardization of available datasets. DL methods are beginning to emerge, but their wider use in chronic stress detection is still constrained by limited dataset size, session-based labeling, and the absence of standardized longitudinal benchmarks.

The field also continues to face several methodological challenges, including ambiguity in stress labeling, limited longitudinal validation, circadian and contextual confounding, inter-individual physiological variability, and weak external generalization. Progress will depend on the development of harmonized longitudinal datasets, biologically informed multimodal modeling, adaptive personalization strategies, and more rigorous subject-independent evaluation.

Future advances should not be judged by classification accuracy alone. Greater emphasis will also be needed on biological interpretability, explainability, and clinical validation. To be useful in practice, chronic stress detection systems should demonstrate value beyond self-report measures and show relevance for occupational health, mental health, and preventive medicine. With stronger physiological grounding and more robust, interpretable AI frameworks, the field may move closer to clinically meaningful and scalable chronic stress monitoring.

## Figures and Tables

**Table 1:** Search concept groups and associated keywords for AI-based chronic stress detection.

Component	Search Content
Indexing Databases	Scopus, PubMed, Web of Science (WoS)
Group I: Stress Type	“chronic stress” OR “long-term stress” OR “persistent stress” OR “prolonged stress” OR “allostatic load”
Group II: Inferential Task	Detection OR Identification OR Recognition OR Assessment OR Monitoring OR Classification
Group III: Modalities	EEG OR Electroencephalography OR fNIRS OR fMRI OR ECG OR Electrocardiography OR HRV OR “heart rate variability” OR EDA OR “electrodermal activity” OR GSR OR PPG OR photoplethysmography OR “wearable sensors”
Boolean Logic	(Group I) AND (Group II) AND (Group III)
Search Strategy	Integrated Query: (Group I) AND (Group II) AND (Group III) Granular Refinement: If integrated strings exceeded database character limits, queries were decomposed into individual signals (e.g., Group I AND Group II AND [Signal]).
Inclusion Focus	Peer-reviewed studies reporting AI-based, ML, or automated computational approaches for chronic stress inference.

**Table 2:** Summary of physiological modalities and AI methods in chronic stress detection.

Ref	Sample Size (*n*)	Modality	Inferential Paradigm	Extracted Features	Label Type	AI Model	Validation Scheme	Notes
[29]	26	EEG	Resting	Fractal dimension, Gaussian mixture features of EEG spectrograms, and magnitude-squared coherence	PSS-14 items	SVM, k-NN	Subject-independent: LOSO	Chronic mental stress classification using PSS-based labels
[30]	40	Multimodal (EEG + ECG + EDA)	Resting	Spectral power and statistical features from HRV and EDA	PSS-10 items	MLP, SVM, NB	Subject-independent: LOSO	Feature-level multimodal fusion
[31]	39	PPG	Longitudinal wearable	HRV (RMSSD, AVNN, SDNN, etc)	PSS + Personal Score (1 to 10)	SVR, RF, MLP, etc.	Subject-dependent: 80/20 train/test split	Two-stage wearable monitoring framework
[32]	28	EEG (single-channel)	Resting	Time-domain statistical features, power spectrum, wavelet energy	PSS	SVM	Subject-dependent: 70/30 train/test split	Feasibility of reduced-channel EEG
[33]	64	EDA + activity	Longitudinal	Activity magnitude, activity state, and EDA-derived features	PSS-10 items	SVR	Subject-dependent: 5-fold Nested CV	Temporal aggregation in wearable monitoring
[34]	56	HRV + ECG + EDA	Reactivity	HRV modulation and EDA amplitude	PSS-10, CD-RISC	Statistical analysis	n/a	Acute stress response profiling
[35]	104	ECG + acceleration	Longitudinal	Time-slot HRV features	PSS, SDS, SAS	Fully connected NN	Subject-dependent: appx. 6:1 train/val split	Ambulatory physiological monitoring
[36]	28	Multimodal (EEG + GSR + PPG)	Resting	Time-domain EEG, GSR, and PPG features	PSS	SVM, NB, MLP	Subject-dependent: 10-fold CV	PSS-based group classification
[37]	28	EEG headband	Resting + Task	Band power, correlation, asymmetry	PSS-10 items	SVM, NB, MLP	Subject-dependent: 90/10 train/test split	Commercial wearable EEG system
[38]	46	EEG	Reactivity	Differential entropy (DE)	PSS-10 items	CNN-based context-aware model	Subject-independent: Participant-level split	Context-aware stress classification
[39]	33	EEG	Resting	Band power, asymmetry	PSS-10, psychologist labelling	SVM, NB, k-NN, LR, MLP	Subject-dependent: 10-fold CV	Long-term stress labeling
[40]	38	Multimodal Smartwatch	Wearable	HR, HRV, RR, sleep-related features	PSS, DASS	LightGBM	Subject-independent: 1 participant = 1 epoch of data	Smartwatch-based chronic stress framework
[41]	n/a	Wearable multimodal (HRV + GSR + EDA)	Longitudinal	HRV, GSR, and EDA features	PSS	ML classifiers	Subject-independent: LOSO CV	Real-time wearable stress monitoring
[42]	39	EEG + ECG	Reactivity	Band power, asymmetry, HRV, and statistical features	PSS, TICS	ExtraTree, k-NN, RF	Subject-dependent: 70/30 train/test split	Cross-modal comparison of resting and reactivity conditions
[43]	131	Wearable PPG	Longitudinal	tsfresh HRV features	CPSS-14 items	RF, SVM	Subject-independent: Individual level aggregation	Consumer wearable implementation
[51]	98	EEG	Resting	Functional connectivity	MBI-GS, BDI	Statistical analysis	n/a	Burnout as a proxy of chronic stress
[52]	33	EEG + ECG + cortisol	Resting	Beta-band power, SDNN, cortisol	SRI	Statistical analysis	n/a	Multimodal integration including endocrine marker
[62]	58	Pupil Diameter + HR + EDA + Respiration	Reactivity	Task-evoked pupil diameter, HR, pulse wave amplitude, RR	STAI	LR, NB, MLP, RF, and K-star	Subject-independent: Subject-wise 10 fold CV	Blunted physiological reactivity
[66]	14	EEG	Reactivity (Stroop)	Task-related alpha, beta, and gamma modulation	PSS-10, TICS	SVM, RF, LR	Subject-dependent: k-fold CV	EEG-based chronic stress classification
[66]	68	HRV	Resting	HRV features (SDNN, RMSSD, LF/HF, etc.)	SRI	LR	Subject-dependent: 5-fold CV	Self-reported stress stratification
[68]	62	PPG	Wearable	Statistical features from PPG	n/a	LR, RF, ExtraTree, SVM, MLP	Subject-dependent: Subject-wise 75/25 Hold-out	Older adults cohort
[69]	30	HR + EDA + Temperature	Resting	HR, GSR, Temperature	DASS	k-means clustering	Subject-dependent: unsupervised clustering	Unsupervised physiological grouping
[71]	18	EEG	Resting	Band power, coherence, entropy	DASS	SVM, LDA	Subject-dependent: 5-fold & LOO CV with SMOTE	Sequential backward features selection

Note: CV: Cross Validation; SDS: self-rating depression scale; SAS: self-rating anxiety scale; TICS: Trier Inventory for Chronic Stress; CPSS: Chinese version of the Perceived Stress Scale; STAI: State-Trait Anxiety Inventory; SRI: Stress Response Inventory.

## Data Availability

No new data were created or analyzed in this study. Data sharing is not applicable to this article.

## Abbreviations

The following abbreviations are used in this manuscript:

AI	Artificial Intelligence
BDI	Beck Depression Inventory
CD-RISC	Connor-Davidson Resilience Scale
CNN	Convolutional Neural Network
CPSS	Chinese Perceived Stress Scale
DASS	Depression Anxiety Stress Scale
DL	Deep Learning
ECG	Electrocardiography
EDA	Electrodermal Activity
EEG	Electroencephalography
fMRI	Functional Magnetic Resonance Imaging
GSR	Galvanic Skin Response
HCC	Hair Cortisol Concentration
HF	High Frequency
HPA	Hypothalamic–Pituitary–Adrenal
HRV	Heart Rate Variability
k-NN	k-Nearest Neighbors
LDA	Linear Discriminant Analysis
LF	Low Frequency
LOSO	Leave-One-Subject-Out (validation)
LR	Logistic Regression
LSTM	Long Short-Term Memory
MBI-GS	Maslach Burnout Inventory—General Survey
MEG	Magnetoencephalography
ML	Machine Learning
MLP	Multilayer Perceptron
MRI	Magnetic Resonance Imaging
NB	Naïve Bayes
PPG	Photoplethysmography
PSS	Perceived Stress Scale
RF	Random Forest
RMSSD	Root Mean Square of Successive Difference
SAM	Sympathetic-Adrenomedullary
SDNN	Standard Deviation of Normal-to-Normal Intervals
SRI	Stress Response Inventory
SVM	Support Vector Machine
SVR	Support Vector Regression
TICS	Trier Inventory for Chronic Stress
